# Role of compliant mechanics and motor control in hopping - from human to robot

**DOI:** 10.1038/s41598-024-57149-0

**Published:** 2024-03-21

**Authors:** Aida Mohammadi Nejad Rashty, Maziar A. Sharbafi, Omid Mohseni, André Seyfarth

**Affiliations:** Lauflabor Locomotion Laboratory, Institute of Sport Science and Centre for Cognitive Science, Technical University of Darmstadt, Darmstadt, 64289 Germany

**Keywords:** Electrical and electronic engineering, Mechanical engineering

## Abstract

Compliant leg function found during bouncy gaits in humans and animals can be considered a role model for designing and controlling bioinspired robots and assistive devices. The human musculoskeletal design and control differ from distal to proximal joints in the leg. The specific mechanical properties of different leg parts could simplify motor control, e.g., by taking advantage of passive body dynamics. This control embodiment is complemented by neural reflex circuitries shaping human motor control. This study investigates the contribution of specific passive and active properties at different leg joint levels in human hopping at different hopping frequencies. We analyze the kinematics and kinetics of human leg joints to design and control a bioinspired hopping robot. In addition, this robot is used as a test rig to validate the identified concepts from human hopping. We found that the more distal the joint, the higher the possibility of benefit from passive compliant leg structures. A passive elastic element nicely describes the ankle joint function. In contrast, a more significant contribution to energy management using an active element (e.g., by feedback control) is predicted for the knee and hip joints. The ankle and knee joints are the key contributors to adjusting hopping frequency. Humans can speed up hopping by increasing ankle stiffness and tuning corresponding knee control parameters. We found that the force-modulated compliance (FMC) as an abstract reflex-based control beside a fixed spring can predict human knee torque-angle patterns at different frequencies. These developed bioinspired models for ankle and knee joints were applied to design and control the EPA-hopper-II robot. The experimental results support our biomechanical findings while indicating potential robot improvements. Based on the proposed model and the robot’s experimental results, passive compliant elements (e.g. tendons) have a larger capacity to contribute to the distal joint function compared to proximal joints. With the use of more compliant elements in the distal joint, a larger contribution to managing energy changes is observed in the upper joints.

## Introduction

Biological systems have developed over thousands of years of evolution. Legged locomotion is one of the advanced capabilities of these evolutionary systems, which is far beyond the capabilities of legged machines. Learning how the mechanics and control of biological locomotor systems work could allow the building of more agile and efficient legged robots and more supportive assistive systems. In that respect, human experiments, data analyses, and developing models to predict similar behavior using the identified movement principles are the typical approach. However, the gap between models and the real world always challenges the sufficiency of the simulations to prove the developed hypotheses. Previously, we designed the BioBiped as a bioinspired biped robot^1^ with muscle-like actuation systems to verify the identified features of human biarticular muscles for different locomotion subfunctions. The lack of accessibility to tune stiffness in the series elastic actuators or springs, which were used to model leg muscle groups in the BioBiped, hindered the separate investigation of the role of mechanics and control without changing the robot hardware (e.g., springs). By introducing the electric-pneumatic actuator (EPA)^2^, we provided direct access to control (using electric motors) and adjustable mechanical compliance (through pneumatic artificial muscles (PAM)). Thus, we follow the approach of using hardware systems to validate biomechanical findings: first, by tuning the mechanical compliance of the hybrid actuation system for the EPA-Hopper-II based on the findings from human hopping experiments. Second, by applying a controller which closely replicates human motor control to generate robot hopping at different frequencies.

Humans are able to perform a variety of movement tasks, rapidly change their gait, and adapt to different environmental conditions^3–10^. Comparing walking at different speeds shows that the largest power changes are observed at the knee and then at the hip joint, while the ankle power does not change considerably^3,11^. In running, knee joint stiffness is increased, while ankle joint stiffness remains constant as running velocity increases^12–14^. In hopping, stiffness and damping profiles of the leg joints during the ground-contact phase can be predicted by a linear variable stiffness and constant damping; while different joint stiffness will increase with increasing hopping frequency^7^.

In human locomotion, the leg dynamics are shaped by the action of the muscle-tendon systems across the major leg joints (ankle, knee, and hip). In human hopping, next to the dominant role of the ankle joint on leg dynamics^15^, the contributions of the knee and hip joints increase while hopping higher or slower (lower frequency)^16^. Depending on the muscle activation and intrinsic muscle dynamics (preflex function^17^), the stiffness adapts with the frequency and amplitude of the movement (changing hopping height)^18^. It is known that body mechanics (e.g., compliance) and motor control complement each other to perform a movement task optimally^19^. Still, it needs to be clarified how the motor control achieves the tuning of joint function, like increasing joint stiffness. Different muscle control mechanisms have been proposed in the literature, e.g., reflex-based control using force feedback or length (stretch) feedback^20,21^.

The main goal of this study is to investigate the role of motor control and passive mechanics in human hopping at different frequencies as a template model to design and control a bioinspired hopper robot. We hypothesize that joint compliance changes with hopping frequency and is composed of both adjustable passive compliance (e.g., the recruitment of titin filaments in muscles, Ref.^22^) and modulated muscle stiffness based on sensory feedback^20,23,24^. We expect different contributions of sensory feedback control to adjust joint stiffness at the ankle, knee, and hip. Based on our previous studies^25^, we hypothesize that the more distal the joint (more distant to the center of mass (CoM)), the higher the capacity to benefit from the passive elasticity. In contrast, a higher contribution to energy management (e.g., with reflex control) is expected for the more proximal joints (closer to the CoM).

Biomechanical template models such as spring-loaded inverted pendulum (SLIP) model^18,26,27^ are well-established concepts for explaining human and animal locomotion. This approach utilizes passive elements in conservative models. A more detailed model level (anchor^28^) was introduced in neuromuscular reflex-based gait models^20^. In this approach, muscle dynamics and neural feedback generate the same spring-like leg behavior as described in the SLIP model. Finding a realistic modeling approach following the template-anchor concept^28^ in biomechanics and robotic applications^29^ is still a challenge. One recent step in this direction is the force-modulated compliance (FMC) concept for legged locomotion control^30–33^. In this method, the antagonistic muscles are represented by a spring while the ground reaction force (GRF) is used as an activation signal to tune its stiffness. Such an application of the GRF to adjust the joint (or muscle) stiffness in the FMC was introduced as a template-based approach that benefits from sensory feedback^30^. This method bridges biomechanical template models and neuromuscular anchor models. It can successfully describe joint, and muscle function adaptations in various gait conditions (e.g., walking speeds)^34^. Here, we follow this approach as an abstract representation of human reflex control. Applying this approach to analyze human hopping will help us improve our understanding of human motor control.

**Figure 1:**
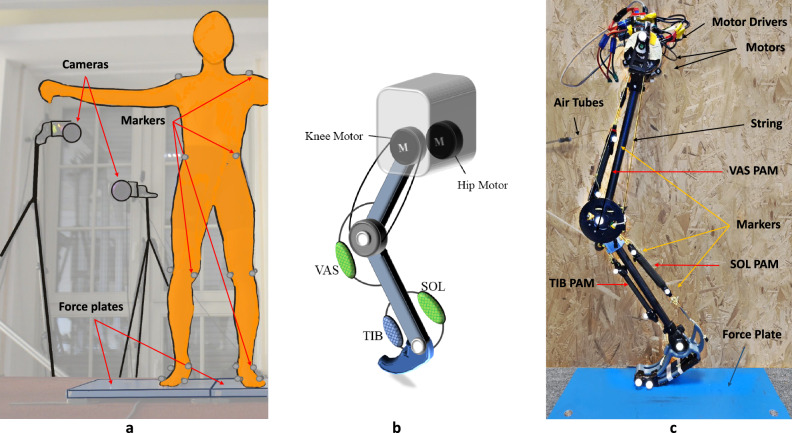
From human to robot hopping, (**a**) Human hopping experimental setup: the motion capture cameras, markers, and force plates. The subject stands in the static position to capture the static trial, which is used to scale the model in OpenSim, (**b**) Bio-inspired actuator arrangement schematic model of EPA-Hopper-II with VAS, SOL, and TIB PAMs, and (**c**) EPA-Hopper-II Experimental setup.

Inspired by the inverse biomimetics concept introduced in^35^, we develop a physical system that can be used to affirm the biomechanical hypotheses. Our bioinspired EPA-Hopper-II robot will be used to demonstrate the reverse engineering approach for a biological locomotor system. The human hopping results shaped the design of the robot. The experimental analysis of hopping reflects the hybrid architecture of the EPA design, namely tunable compliance (pneumatic artificial muscles) and reflex-inspired control (FMC concept). After analyzing human hopping, we examine the validity of the identified mechanical design (e.g., passive compliance) and motor control (e.g., FMC) principles in robotic experiments (Fig. 1).

## Results

This section includes human hopping analyses and results of implementing the identified mechanics and motor control on the EPA-Hopper-II robot.

### Human gait analyses

The human hopping analysis comprises three levels: (1) Mechanical effects, characterized by elasticity coefficient and residual torque ratio, (2) Energy injection and absorption characterized by work-loop to envisage active motor control e.g., using FMC, and (3) Co-adaptation of mechanics and control to generate hopping at different frequencies.

**Figure 2:**
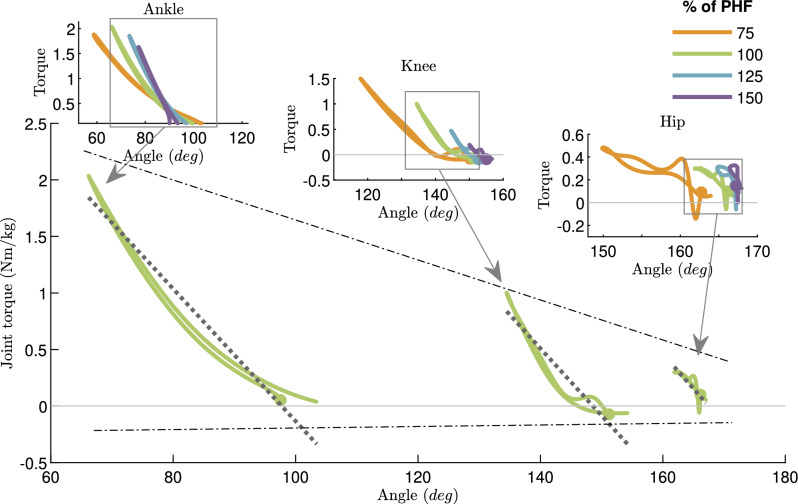
Torque-angle relationship in different leg joints in hopping. The graphs for preferred hopping frequency (PHF) and their approximation with a linear relation (dotted line) are shown in the bottom figure. The patterns for four hopping frequencies (75, 100, 125, and 150 % of PHF) in each leg joint are shown in the top graphs. Small circles indicate the touch-down moments.

Figure 2 illustrates the torque-angle characteristics of different joints at preferred hopping frequency (PHF) and 75%, 125%, and 150% of PHF (shown in small graphs on top). The linear patterns in the ankle joint demonstrate the spring-like behavior at different frequencies. The knee joint behavior does show not only nonlinearity but also the necessity of energy injection/absorbtion depicted in the work-loop^36,37^ graph (more detailed graphs are illustrated in Fig. S3 in the Supplementary Information). At the hip joint, more complex behavior is observed, which might result from more complicated motor control. Figure 2 also shows that the torque magnitude decreases from the distal to the proximal joint. Dividing the average joint torques by the summation of average torques of the three joints at preferred hopping frequency (PHF) will result in 62 %, 25 % and 13 % for ankle, knee, and hip joint, respectively. These ratios reveal the least contribution of the hip joint in preferred hopping. Statistical analysis (one-way analysis of variance (ANOVA)) is used to compare joint different properties. The ankle torque (most distal) remains almost constant (*p*-value = 0.28) for the first 3 hopping frequencies, and a slight decrease is observed in the fastest frequency (*p*-value $< 0.05$). With increasing hopping frequency, the knee torque decreases (*p*-value $< 0.05$), which shows its higher effect in lower hopping frequencies. The hip torque is similar (*p*-value = 0.46) in the PHF and higher frequencies, while there is a slight increase (*p*-value $< 0.05$) at 75 % PHF.

**Figure 3:**
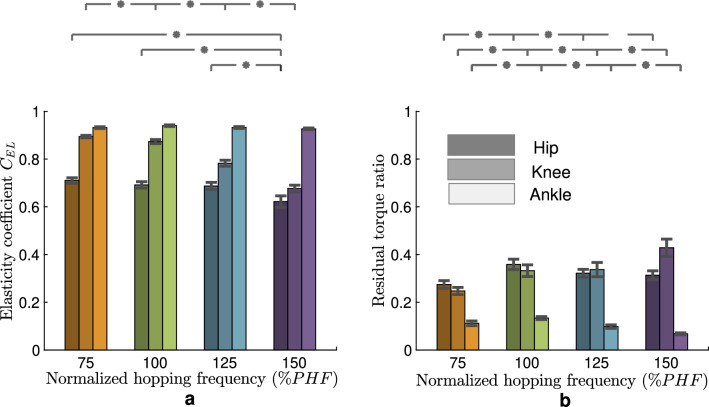
Quantifying the joint elastic behavior. (**a**) The elasticity coefficient ${C_{EL}}$ in different leg joints for different hopping frequencies, and (**b**) Residual torque ratio: the ratio of residual torque to the joint torque for different hopping frequencies. The residual torque is the difference between the joint torque and torque predicted by the elastic term (linear spring model). The standard error for each value is shown as an error bar. The significant difference between different values in different frequencies for different joints is shown with small stars.

The spring-like behavior could be quantified by elasticity coefficient ($C_{EL}$)^20,25^, and it serves as a metric of how closely the joint approximates perfectly elastic behavior. The maximum elasticity coefficient is 1, belonging to springs regardless of their stiffness or nonlinearity. Figure 3a displays elasticity coefficients for different joints at different frequencies. The ankle joint shows a consistent highly elastic behavior characterized by elasticity coefficient $C_{EL} > 0.92$, which is not significantly different for different frequencies (*p*-value = 0.42). The elasticity coefficient in the knee joint decreases (from 0.89 to 0.67) with increasing hopping frequency (*p*-value $< 0.05$). The knee behaves less elastic than the ankle but still has spring-like properties, especially in slow and preferred hopping frequencies. The lowest elasticity (less than 0.7) is found at the hip joint, while the values are constant for the first three frequencies and decrease to 0.63 for the fastest hopping (the *p*-value of about 1 indicates that the elasticity coefficients in first 3 frequencies are the same and the fastest frequency is significantly different from others (*p*-value $< 0.05$)). Figure 2 shows how the nonlinear torque-angle behavior at different joints can be approximated by a straight line. Considering joint elastic behavior (based on high $C_{EL}$ values), a better fitting is expected for the ankle joint. For each trial of different subjects’ hopping at a specific frequency, we fitted the line and calculated the $R^2$ coefficient of determination for approximating the joint torques. Table 1 presents the average $R^2$ values for different joints at each frequency. Finding $R^2$ > 95% for the ankle joint demonstrates that a linear spring with specific stiffness suffices to predict this joint torque at different frequencies.

**Table 1:** Prediction of human joint torque using different methods.

Joint	Frequency	Spring	FMC	FMC+Spring
	(% of PHF)	Coefficient of determination ${R^2}$ (%)		
Hip	75	62	57	65
	100	35	27	48
	125	36	29	52
	150	27	48	69
Knee	75	91	98	98
	100	85	94	97
	125	80	80	89
	150	68	55	76
Ankle	75	96	97	99
	100	95	98	99
	125	96	98	99
	150	97	98	99

The $R^2$ coefficient of determination between the real values and the prediction with these three methods, passive *spring*, *FMC*, and a combination of FMC and passive spring (*FMC+spring*) are presented for four frequencies (75, 100, 125, and 150% of PHF).

In order to improve our understanding of the role of the passive elastic element at each joint, we computed the residual torque ratio through the following process. Initially, we determined the anticipated torque by fitting the joint’s torque-angle relationship with the best-fitted linear spring model at the particular frequency. Subsequently, we defined the residual torque as the difference between this spring torque and the actual joint torque. The residual torque ratio (Fig. 3b) was derived by dividing this residual torque by the joint torque and then averaging it over the gait cycle. At the ankle joint, this averaged required active torque is less than 15 %, decreasing with frequency increase from PHF (*p*-value $< 0.05$). The pattern is similar to the ankle at the hip joint. The percentage of residual torque is between 30–35% for 100–150%PHF, decreasing with increasing frequency (*p*-value $< 0.05$). At the knee joint, there is an increasing tendency to use active control with increasing hopping frequency (*p*-value $< 0.05$), and the maximum value occurs at 150 % PHF (44.8 %). In the Supplementary Information, the same calculations have been done for the nonlinear springs. Figure S1 demonstrates that with a more complex nonlinear spring (e.g., a cubic polynomial), the residual torque will be reduced for all joints, but the patterns of having much higher residual torque ratio at proximal joints are similar. The residual torque of the hip joint is still more than 25% for all frequencies. For the knee joint, this ratio increases from 10 to 34% by increasing the hopping frequency, while for the ankle joint, it is less than 5% for all frequencies.

In order to better understand the contribution of passive and active elements at each joint, we analyzed the work-loop and calculated the net positive and negative energy at each joint. The work-loops for different hopping frequencies for the knee joint are shown in Fig. S3 in the Supplementary Information. We divided the work-loop to the total injected and absorbed energy and defined this as a normalized work-loop. Our analysis revealed that the normalized work-loop is less than 5% for the ankle joint, and increases from 10% to 33% for the knee joint. For the hip joint, the normalized work-loop is between 16% and 20%, as shown in Fig. S2 in the Supplementary Information. This indicates that the ratio of energy that cannot be communicated by the passive joint to the total contribution of joint energy is much higher at the proximal joint.

Our analysis of the normalized work-loop, combined with low elasticity coefficients and higher residual torque ratio in proximal joints, suggests the need for active energy modulation in these joints. In the second step, we developed an active model to predict hip and knee torques, in which we examined the FMC control method. As shown in Table 1, the FMC formulation could significantly improve the prediction of the torques at the knee and hip joints. The FMC also has the potential to nicely predict the work-loop at the knee joint, which is not possible with linear or nonlinear springs, as shown in Fig. S4 in the Supplementary Information. The *FMC+Spring* model could nicely approximate the knee torque for 75-125% PHF, and the prediction for 150% is also acceptable. Despite the significant improvement in hip torque prediction, a more complicated controller is needed. For the ankle joint, the FMC can increase the correlation 2-3 % to reach $R^2\ge \,$ 98 %, and the *FMC+Spring* model provides 99 % correlation. In all cases, combining active and passive compliance could improve the results. However, due to the high precision of ankle torque replication with the linear spring model, the minimalistic modeling approach prescribes a passive elastic model for this joint. The *FMC+Spring* is the preferred model to replicate human knee joint behavior at different frequencies. Hereafter, we focus on the ankle and knee joints for the following reasons: 1- the proposed minimalistic active-passive model is not sufficient for predicting hip joint torque-angle behavior, 2- the hip contribution is less than the other two joints, 3- the hip is more responsible for upper body balancing, which cannot be verified with our robotic setup, as the robot trunk is constrained to be upright.

The last step of our human gait analyses focuses on identifying the mechanics and control adaptation procedure that generates hopping at different frequencies. Tuning the stiffness and rest length of the physical and virtual (through FMC) springs is the strategy for adapting the hopping frequency.

**Figure 4:**
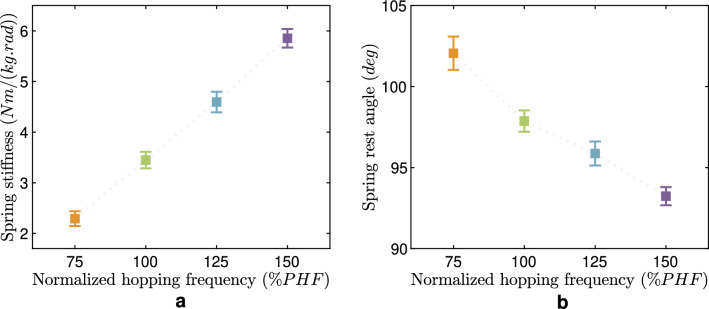
Parameters of the passive spring model to predict human ankle torque in hopping at different frequencies. (**a**) Spring stiffness and (**b**) Spring rest angle for all hopping frequencies. The mean value and standard error among the six subjects are shown. The identified values for spring parameters at each frequency are statistically different (both *p*-values for stiffness and rest angle are less than 0.05) from the corresponding values in other frequencies. The dotted line between data points is plotted to illustrate the trend of changes and does not mean interpolation.

Figure 4 shows the calculated ankle joint stiffness and rest angle for different hopping frequencies. The spring stiffness increases, and the rest angle decreases with increasing hopping frequency. We ran the one-way ANOVA that reveals the significance of the changes in the ankle spring parameters. The identified values for spring parameters at each frequency are statistically different (*p*-values $< 0.05$ for stiffness and rest angle) from the corresponding values in other frequencies. Thus, the ankle stiffness needs to be tuned to change the hopping frequency, which motivates having PAMs (without motor) as an easy-to-adjust elastic element that can be used as passive compliance in the robot’s ankle joint. Tuning the PAM pressure in the ankle antagonistic artificial muscles (Soleus (SOL) and Tibialis anterior (TIB)) can adjust the stiffness and rest angle as prescribed.

**Figure 5:**
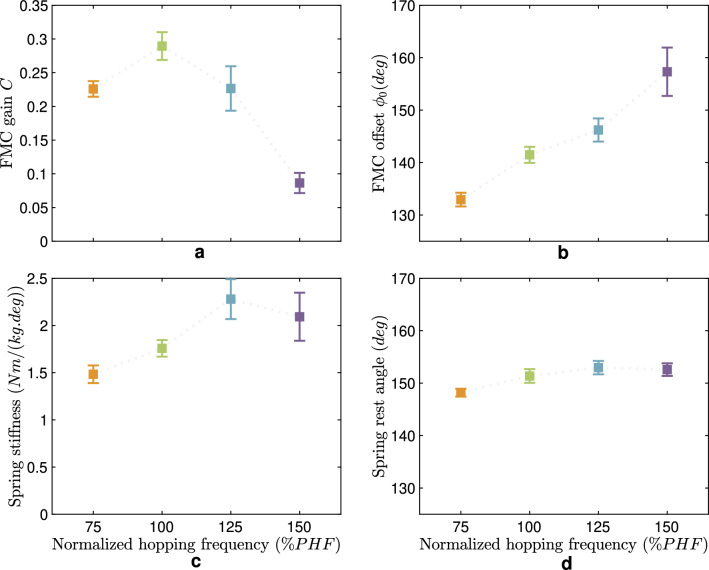
Parameters of the *FMC+Spring* model (FMC and a passive parallel spring) to predict human knee torque in hopping at different frequencies. (**a**) FMC gain *C* and (**b**) Rest angle $\theta ^f_0$ are the parameters of FMC, which are significantly different for different hopping frequencies (*p*-values are less than 0.05). The values of the parallel spring (**c**) Stiffness and (**d**) Rest angle are not statistically significantly different (*p*-values are bigger than 0.05). The mean value and standard error among the six subjects are shown. The dotted line between data points is plotted to illustrate the trend of changes and does not mean interpolation.

Figure 5 illustrates the changes in the parameters of the knee spring (passive mechanical element) and the FMC (active reflex-based control) in different frequencies. As is evident, the FMC gain *C* decreases (*p*-value $< 0.05$) with increasing hopping frequencies, except for the lowest frequency, whose normalized stiffness *C* is less than the corresponding value in PHF. An increasing pattern versus hopping frequency is observed for the FMC offset $\theta ^f_0$ (*p*-value $< 0.05$). The parallel spring stiffness and rest angle exhibit an increasing trend except for the fastest frequency. Although these parameters follow a general pattern of growth, the coefficients experience differing rates of increase. Our statistical analyses indicate no statistically significant difference between spring stiffness and rest angle in different hopping frequencies (*p*-value > 0.05).

### Robot experiments analyses

Based on our findings from the human data analyses, we adapt the EPA-Hopper-II with PAMs as passive actuators at the ankle joint and FMC in parallel with the knee PAM, which represents the Vastus (VAS) muscle in the human knee structure. Two PAMs in the ankle joints represent the SOL and TIB muscles in the human body. Due to statistical analyses of the human ankle joint in hopping, we simultaneously increased (or decreased) ankle antagonistic PAMs’ pressures to increase (or decrease) stiffness while tuning the rest angle.

At the knee joint, we kept the PAM’s pressure constant and changed the hopping frequency by changing FMC parameters. By combining knee and ankle strategies to tune the hopping frequency derived from human data analyses, we achieved a comparable frequency tuning in EPA-Hopper-II hopping. The stable hopping frequencies are $2.3,\,\,2.5,\,\,2.7,\,\,and\,\,2.9\,Hz$.

**Figure 6:**
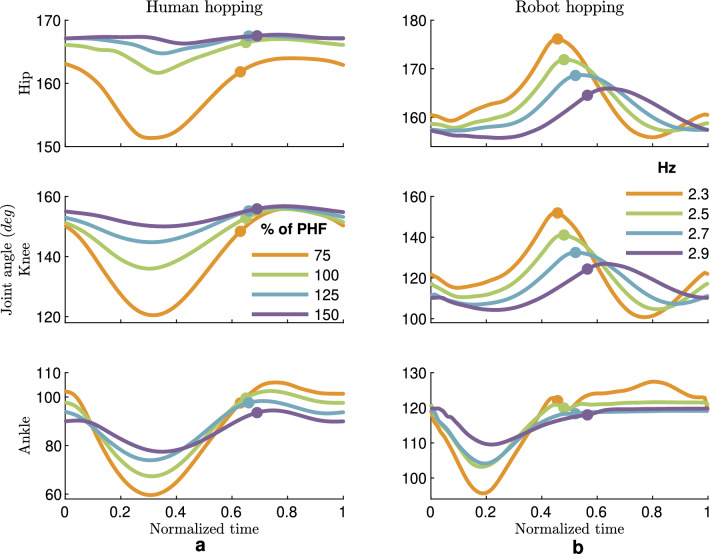
Comparison of human and robot leg joint angles (during a hopping cycle). In both (**a**) Human and (**b**) Robot hopping, the range of motion in all leg joints decreases with increasing hopping frequency. The average data of all subjects for each frequency is shown. The robot data is the average value for each frequency. The Small circles indicate the take-off moments.

In Fig. 6, human and robot joint kinematic behaviors are compared. The range of motion in humans and robots increases by lowering the frequency. All joints move less (the difference between the maximum flexion and extension) when the frequency grows, so we have the minimum movement of all leg segments at the fastest frequency. The ankle joints of the robot and human have the highest similarities at different frequencies. In the stance phase, the joint motion range also shrinks, moving from distal to proximal joints in both human and robot experiments. More symmetric patterns are observed in different human joint angles during either stance or flight phases. For example, robot knee extension in the upward movement is more pronounced than knee flexion.

**Figure 7:**
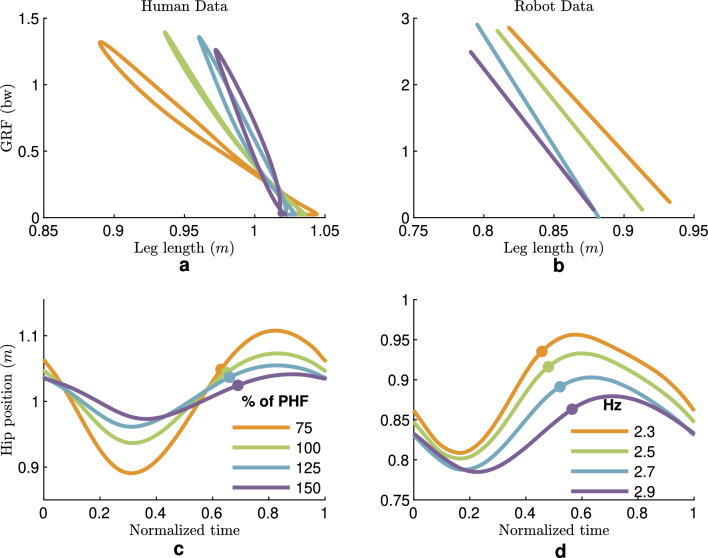
Comparison of human and robot leg behavior. (**a**) Leg force-length curve of human hopping, (**b**) A linear estimation of the force-length curve in robot hopping (for better readability), (**c**) Human hip (hopping height is 4.9, 2.4, 1.6, and 1 cm for 75-150% PHF, respectively), and (**d**) Robot hip position (hopping height is 2.1, 1.7, 1.6, and 1.2 cm for 2.3-2.9 Hz, respectively) The average data of all subjects for each frequency is shown. The robot data is the average value of all hops for each frequency. Small circles indicate the take-off moments.

A comparison between human and robot leg behavior is presented in Fig. 7. The leg stiffness needs to be higher to increase hopping frequency while the leg rest length slightly diminishes. This is observed in both human and robot hopping, except for the robot leg stiffness at the fastest frequency.

Figure 7 shows hip height which is an indicator of body movement. In both experiments, the hip movement is decreased with increasing hopping frequency. The maximum excursion in the hip position is observed in the lowest hopping frequency in human and robot experiments. In human hopping, the hip movement is more symmetric in the stance phase compared to robot hip movement, which is longer in the extension phase.

These comparable behaviors in humans and robots at the leg level are achieved by simply tuning physical and control parameters in the robot’s knee and ankle joints.

## Discussion

In this paper, we studied human body mechanics and control adaptations at the leg and joint level for different hopping frequencies. The main findings are as follows: (1) *Simultaneous mechanics and control adjustment:* a specific tuning of mechanical and control parameters are found for each movement task (hopping at a given frequency). (2) *Different contributions of mechanical and control parameters to generate the movement at different joints:* (2a) higher contribution of (passive) mechanical elements at more distal joints, and (2b) higher contribution of the controlled joint dynamics (e.g., using FMC) at more proximal joints. (3) *Dominant roles of knee and ankle joints in shaping leg behavior in hopping.*

These design and control findings are verified in the real world using the bioinspired hopping robot setup. The lessons learned from robot investigations and experiments are (4) *Sufficiency of adjustable passive elasticity for replicating human-like ankle joint behavior in hopping while needing actuators for Knee and hip joint functions.* (5) *Minimal control with a combination of passive and active element control:* for hopping at different frequencies, different ankle stiffness values were observed, which suggests ankle stiffness adjustment for frequency tuning and active control of the knee joint for energy management e.g., by reflex control. (6) *Need for further investigations in robot hopping.* This study did not investigate joint contributions for balancing. Biarticular arrangement and damping effect of providing further synchronicity between different joints’ movements and efficient energy management need to be explored in updated robot designs.

### Simultaneous mechanics and control adjustment

Leg elasticity shapes bouncing gaits such as human hopping. Our analysis shows that this essential characteristic results from a specific combination of passive (mechanical) and active (controlled) compliance at different leg joints. Humans benefit from both active and passive characteristics of muscle dynamics to achieve efficient and robust hopping^38^. This mechanical and controlled properties recruitment varies for different joints and is required to tune hopping frequency. For example, Monte et al. showed that in hopping, the knee transitions from spring-like to strut-like function as frequency increases while the ankle remains mainly spring-like with negligible damper and motor-like effects^39^. Hence, our findings can be used for robot design and control to replicate human hopping. This is in continuation of our previous studies on developing theoretical methods for concurrent design and control of legged robot locomotion^40,41^. The hybrid actuation concept in EPA^2^ enables us to benefit from this simultaneous mechanic and control adjustment.

To predict human hopping, we tested minimalistic models of (linear passive and modulated springs) to represent these features at the different leg joints. As shown in Table 1, the proposed models could well describe the observed torque-angle behavior at knee and ankle joints. This model was also capable of predicting hip torque control at low frequencies. Despite significant improvement in hip behavior prediction precision (40-130 % increase) by adding the FMC to the spring for moderate to fast hopping, the resulting prediction accuracy is less than 50 % and reveals the need for model extension.

### Role of mechanics and control at different joints

Both control and mechanical characteristics play essential roles in hopping control at different joints, while the contribution of each passive and active element varies from distal to proximal joints. As shown in Fig. 3a, the highest and most consistent elastic behavior is observed in the ankle joint, the closest joint interacting with the ground. This means that our body relies more on mechanical properties for immediate interaction with the environment^42^. The lowest contribution of active control can also be observed in the residual torque ratio at the ankle joint (Fig. 3b). Table 1 shows the sufficiency of a linear spring with specific stiffness and rest angle to predict human-like ankle torque-angle behavior with $R^2$ values higher than 95% for hopping at each frequency. Notwithstanding, the identified spring parameters are different at various hopping frequencies, as shown in Fig. 4. Ankle stiffness increases (*p*-value $< 0.05$) with hopping frequency while the rest angle slightly decreases (*p*-value $< 0.05$), as found in previous studies^7,16^.

These results also suggest using pre-tuned PAMs for the ankle joint of the hopping robot. Another alternative could be using clutches to have (quasi-) passive but adjustable stiffness. Such ideas were implemented on assistive devices to support ankle joint with exoskeletons^43–45^ and prostheses^46,47^.

Moving from distal to proximal joints (from ankle to knee), a higher contribution of controlled compliance is found (Fig. 3b). In contrast to the ankle joint, the remaining need for a controlled knee joint function (after reducing the passive spring contribution), quantified by the residual torque ratio, will increase by speeding up the movement. The more elaborated passive spring behavior (e.g., using nonlinear springs) cannot account for the required active contribution of the proximal joint characterized by the residual torque ratio (Fig. S1 in the Supplementary Information). This means that for faster hopping, the knee joint energy absorption/injection modulation role versus its passive behavior is more significant than that of the ankle joint. Similarly, the lower coefficient of elasticity of the knee and hip joints (Fig. 3a) at higher frequencies could indicate a higher active control effort.

Besides the elasticity coefficient and the residual torque ratio, the higher contribution of the knee joint in energy management can be realized by the work-loop analyses, the net required (positive and negative) mechanical work at the joint^36,37^. As shown in Fig. S2, the work-loop of either the knee or ankle joints has the largest values at different frequencies. Dividing the joint work-loop by the total contribution of this joint in injecting and absorbing energy, defined as the normalized work-loop, helps better understand the role of joint active contribution to total energy transfer. The normalized work-loop graphs in this figure demonstrate larger values at the knee and hip joints. Considering both work-loop and normalized work-loop results, the importance of active control at the knee joint will be evident.

Although human motor control cannot be verified using the template-based modeling approaches, they can help better predict emerging behavior and understand the underlying principles. In that respect, the FMC model is a simple representation of the motor control, which can complement the spring-like behavior at the knee joint. The importance of force feedback in locomotion control of biological locomotor systems has already been approved in many studies. For instance, Duysens et al. showed that the leg force continuously adjusts the programmed pattern during movement based on peripheral sensor information^48^. Other studies demonstrated that compensatory leg muscle activation and strength depend on load receptor measurements (such as body weight)^49^. More specifically, leg extensor muscles activation dependency to the load in the stance phase, which is the essence of our FMC control at the knee joint was approved^50^. Studies on locomotion disorders predicted that cyclic leg movement without load feedback could not lead to leg muscle activation^51,52^. From another point of view, the positive force feedback (PFF) concept also supports the importance of contact force measurement^24^. Neuromuscular models, based on the PFF concept, predict different gaits^20,53^. Analyses of different reflex pathways within the sensor-motor map concept highlighted the importance of force feedback in hopping tasks^54^. Finally, the functionality of using the GRF as unique central force feedback for synchronizing different locomotor sub-functions to generate stable hopping was demonstrated in the so-called ”concerted control” concept^55^. Therefore, the FMC control concept can be considered a step towards translating human motor control to be applied for legged robots and assistive devices.

Inspired by all these biological and modeling evidence, we expect the advantages of GRF feedback in generating stable hopping and synchronizing different joints. Interestingly, the required level of energy management can be sufficiently predicted by reducing the normalized stiffness (*C*) and increasing the rest angle ($\theta ^f_0$) of the virtual knee spring (Fig. 5). Here, the passive compliance remains constant (no significant difference in the spring stiffness and rest angle). The main contributor to changing the hopping frequency by the knee joint is the FMC representing an abstracted model of the (force-based) reflex control^20,56^. For hopping at PHF and higher frequencies, reducing the normalized stiffness results in a more compliant knee joint with a reduced hopping height (Fig. 7). Besides, changing the rest angle could provide the negative torque at the early and late stance (before 10% and after 90% of the stance phase), and moderate the positive generated torque throughout the remainder of the stance phase. The adapted knee joint compliance complements stiffer ankle joint behavior to provide the required energy to generate take-off. In other words, humans soften the ankle and stiffen the modulated spring at the knee joint to hop slower. This stiffening controlled spring strategy (in FMC) cannot continue for frequencies below PHF, as it might require too stiff knee for slow hopping, which increases the impact during hopping with higher heights. Then, both rest angle and normalized stiffness ($\theta ^f_0$ and *C*) decrease from 100 to 75 % PHF. We found similar behavior in the robot experiments for the lowest hopping frequency (see details below).

Our modeling approach does not quantify how much of the joint function is created by passive structures but only the capacity of the biological joint function to be generated by elastic elements. Muscles can equally generate close to spring-like joint function under an appropriate neural control (e.g. force- and length feedback circuitries^20^). The share of spring-like joint function to be generated by passive elements compared to muscle function is determined by the need to compensate for energy changes, e.g. in locomotion on uneven terrain^57^. We have conducted human and robot hopping experiments with ground drop perturbations supporting the previous finding in unsteady conditions, which is out of the scope of this study^38,58^. In the following, we elaborate more about the knee and ankle joint functions and corresponding robot experiments in stable hopping at different frequencies.

### Knee and ankle joints shape leg behavior

Considering the leg as an interaction unit between the ground and the body, the relationship between hopping height, frequency, and efficiency can be analyzed regarding leg behavior. Hopping height can be described as the difference between the hip position at take-off and the maximum hip position during the flight phase. Interestingly, big changes in human hopping heights (from 4.9 cm at 75 % PHF to 1 cm at 150 % PHF, observable from the hip position graphs in Fig. 7) will not result in such significant changes in the GRF. The force-length behavior demonstrates the increase in leg stiffness (*p*-value $< 0.05$) and a slight decrease (*p*-value = 0.6) in rest length for achieving faster hopping, which aligns with the ankle joint compliance adjustment. This illustrates how the ankle joint shapes leg behavior. A closer look at the correlation between leg stiffness and joint stiffness clarifies that the dominant joint in determining leg stiffness is the ankle joint. Table S1 in the Supplementary Information shows the correlation between the ankle and leg joint at all frequencies (ranging from 54 to 86% with a *p*-value below 0.05), while there is no correlation to hip stiffness. Although the statistical analyses show *p*-values below 0.05 for the knee joint, the correlation is negative in all frequencies except 75%, which has a 49% correlation. These results are in line with the findings in^10^ showing the correlation only between ankle and leg stiffness for hopping from 2.2 to 3 Hz.

We have also performed sensitivity analyses with a 4-segment model with anthropomorphic segment ratios. We used the joint stiffness and movement excursion identified in human hopping at each frequency to calculate the corresponding leg stiffness. The sensitivity of the leg stiffness variations resulting from separate joint stiffness changes is calculated at different frequencies. Our simulations demonstrate that leg stiffness has the highest sensitivity to ankle joint stiffness, very low sensitivity to the knee joint (in low frequency), and almost no sensitivity to the hip joint stiffness (Fig. S5 in the Supplementary Information). Doubling the ankle stiffness will result in 1.8 to 2 times larger leg stiffness (depending on the hopping frequency). These results are in line with the findings of Farely et al.^15^. Measuring human muscle tendons unit and fascicle length in human hopping also showed the higher contribution of the tendon than the muscles in the ankle joint to recover from perturbations^38^, which support our findings about the dominance of passive elasticity compared to actively controlled behavior in the human ankle joint. Another advantage of the use of elastic elements (e.g. Achilles tendon in the calf muscles) is supporting cushioning of the impact at touch down, an important feature in walking or running over uneven grounds^57^.

As mentioned in the previous section, the analysis of the residual torque ratio, elasticity coefficient, and the work-loop suggests having an actuator at the knee joint. The work-loop graphs for different trials of different subjects at any specific frequency show a large variation, and drawing the work-loop for the grand mean does not illustrate some key characteristics. However, the general behavior at each frequency can be represented by a few typical patterns, shown in Fig. S3 in the Supplementary Information. As can be seen, the knee joint demonstrates negative and positive work-loops, which cannot be generated by passive elastic elements. Active motor control is required to generate such a behavior. Our analysis showed that the FMC could predict the work-loop in most of the trials (see an example in Fig. S4 in the Supplementary Information as an example). It is observed that the nonlinear spring can improve knee torque-angle prediction compared to the linear spring, while it is unable to predict the work-loops. On the other hand, the FMC can compensate for this drawback of the springs by nicely approximating the work-loop. Therefore, a passive compliant element (e.g., a linear spring) beside an appropriate feedback control to inject required energy at the right time (E.g., FMC) could nicely predict the knee joint behavior.

Based on the presented evidence about the role of the ankle and knee joints in generating an elastic leg behavior with the ability to actively contribute to system energy level, this study supports our last hypothesis. In that respect, the leg behavior is shaped mainly by the knee and ankle joints. The role of the hip joint (mainly balancing) is less dominant for shaping the leg force-length behavior in vertical hopping, visible in the torque range shown in Fig. 2. This is in line with previous studies^10,16,59^and the robot experiments (see below).

The reasons for selecting the preferred hopping frequency are not understood as seen in previous studies^15,18^. Another observation from the leg work-loop diagrams (Fig. 7) is that the minimum enclosed area appears at the preferred hopping frequency (100 %PHF). Minimizing the leg work-loop as found here, means a minimum requirement to inject and absorb energy. This could be interpreted as maximum efficiency observed from the elasticity at the preferred hopping frequency. Therefore, humans may prefer frequencies in which they can have the highest efficiency by maximizing elastic leg function.

### Bioinspired joint actuation configuration for hopper robots

The bioinspired structure and actuation design of the EPA-Hopper-II testbed replicates redundant degrees of freedom of the human leg morphology and generates human-like hopping movements. The outcomes of elasticity coefficients, joint contributions in energy management (using work-loop), and the template-based modeling to predict human torque-angle behavior at different joints prescribe (1) a passive but adjustable elastic element for the ankle joint (2) a combination of a passive compliant and active motor for the knee joint and (3) an active motor for the hip joint which could be augmented by a passive compliance. Therefore, we considered three different combinations of electric motors and PAMs for the robot: two antagonistic PAMs for *Ankle*, a combination of an electric motor and an extensor PAM for *Knee*, and an electric motor for the *Hip* joint.

With the antagonistic PAMs at the ankle joint, we can change the stiffness and rest angle of this joint for each frequency by setting the PAM pressures and closing the valves. Therefore, we replicated the patterns found in human hopping to increase/decrease the frequency. As mentioned before, a passive spring cannot explain knee behavior, especially the tuning mechanism to achieve different hopping frequencies. A PAM and an electric motor provide the infrastructure to implement a human-like knee joint design and control on the robot. An electric motor would be sufficient to represent the hip joint actuation in hopping based on the lower elasticity at this joint. This motor can set the hip (correspondingly the leg) angle in the flight phase), but due to our experimental condition, which constrains the hip joint to move only in the vertical direction, the motor was switched off in the stance phase. However, this actuation level is necessary for balancing when the constraint is removed and for hopping freely in the sagittal plane, especially when the trunk has higher inertia than the leg. Recently, we have developed a new version of our hopping robot (called EPA-Jumper) with a more anthropomorphic design, having an extended trunk with comparable (leg to trunk) mass and inertia ratio. Our preliminary experiments showed that adding mono and biarticular PAMs contributing to hip joints could improve balancing (increase the number of hops) but cannot generate stable repetitive hopping, which confirms the necessity of having a hip motor for balancing. Focusing on vertical hopping with lowering the usage of passive dynamics and raising the role of motor control while moving from the distal to the proximal joint is reflected in our robot actuator design. This design can also be traced back to human leg actuation identified in other studies, which reported that during in-place hopping, humans adopt a strut function at the hip^60^, a combination of strut and spring-damper functions at the knee joint^39^, and a spring-like function at the ankle joint^25,39^.

### Minimal control with a combination of passive and active elements

Our robot experiments supported the biological findings. The PAM with a fixed pressure provides sufficient degrees of freedom to mimic the behavior of the passive elastic element at the ankle joint. Thus, we do not need an actuator to regulate the ankle torque continuously. A fixed but tunable elastic element is sufficient to generate steady-state rhythmic movements of the ankle joint in hopping at different frequencies. The comparison of the ankle angle in human and robot for hopping at different frequencies also shows comparable patterns in Fig. 6: (1) higher duty factor (ratio between the stance phase and hopping cycle) and less symmetric ankle angle at fastest hopping, (2) larger joint excursion at lower frequencies, (3) more extended angles at all joints for lower frequencies in the flight phase, (4) symmetric ankle angle patterns in the stance phase, (5) More ankle bending at lower frequencies, (6) Maximum ankle flexion at the middle of the stance phase for the first three frequencies, and (7) low changes in ankle angle in the flight phase.

Using the EPA actuator for the knee joint, the FMC controller can be applied to the motor to manage the energy required for different hopping frequencies while the PAM with a fixed pressure could provide the required passive parallel elasticity. We employed similar patterns for normalized stiffness (*C*) and the rest angle ($\theta ^f_0$) as found in Fig. 5, resulting in hopping frequency changes comparable to humans. In addition, we examined the human experimental findings about tuning *C* and $\theta ^f_0$ to reach the lowest frequency with the robot. For this, besides decreasing $\theta ^f_0$, we increased *C* instead of reducing it (found in human data) from the values set for the 2.5 Hz. We found that this opposite tuning of *C* will increase the hopping frequency (instead of reducing it) with a high impact on the knee joint. Hence, to reach the lowest frequency $2.3\ Hz$, we need to decrease both *C* and $\theta ^f_0$ as found in human hopping data analyses.

We also found differences to human hopping. Higher bending at the knee joint for lower frequencies was also observed in the robot, but the differences are not as pronounced as in human hopping. Such behaviors were partly observed in human hopping on damped surfaces^61^. One potential reason in our robot experiments could relate to the flight to stance transition control, which can be improved by shortening this phase (e.g., from 50 to 5ms. Alternatively, hopping with a straighter leg (larger knee angle) could provide a larger range of knee bending and higher potentials for storing and returning energy at the knee joint, especially at lower frequencies. However, this might cause a higher risk of a large impact on the robot’s knee joint. To avoid that, we selected more bent knee in our experiment, which limited the possibility of knee bending. This enforced constraint (leg configuration) in the robot experiment can be resolved by designing a more human-like leg, e.g., by having higher cushioning at the foot or adding further muscles (PAMs). In both human and robot hopping, the take-off angle is more extended than the touch-down angle, but the difference in the robot is more distinguishable than in humans. In human hopping, the flight phase starts with a small knee extension and then flexion. In contrast, in robot hopping, these two opposite movements will be followed by another knee extension prior to touchdown, except for the highest hopping frequency. This overshoot in the knee angle in the flight phase could be improved either by control or mechanical coupling of different joints. In general, the knee and ankle joints are more synchronized in human hopping than in the robot. This could be because of the lack of the biarticular gastrocnemius (GAS) muscle in the robot. In this study, we did not use GAS in the robot to be able to distinguish between passive and active dynamics at separate joints and to avoid complexity. The effect of GAS can be analyzed by adding a biarticular PAM between the foot and the thigh^62^.

The proposed distal/passive to proximal/active mechanics and motor control paradigm could also yield similar leg behavior. As shown in Fig. 7, increasing stiffness and decreasing rest length of the leg to increase hopping frequency found in human hopping are also observed in the robot, and the only difference is observed in the leg stiffness changes of the fastest hopping. Notably, in robot experiments, the hip controllers are the same for all frequencies. All changes in the leg behavior result from changing the passive elastic behavior of the ankle joint and the controlled compliance at the knee joint (the knee parallel PAM is also fixed in all experiments). The knee motor is the only energy resource in the stance phase in robot hopping, which needs to compensate for all the losses and manage the energy level to enable repetitive hopping. These results support our human experimental findings at the leg level that the leg elastic behavior is shaped by the ankle joint and energy management by the knee joint.

Another representation of the body-to-ground relation could be seen in the CoM movement, which can be approximated by the hip joint in the robot. The hip movement depicted in Fig. 7 reveals similarities between human and robot hopping: (1) The lower the hopping frequency, the more straight the leg at touch down, the more leg compression in the stance phase, and the higher hopping height. (2) The hopping heights and their evolution with frequency are comparable. (3) In fastest hopping, the smallest hopping height with the largest duty factor (compared to other frequencies) is observed. (4) Close to symmetric hip movement in both stance and flight phases.

The leg force-length behavior and related hip excursion are two representations of the interaction between the body and the environment (ground). The identified similarity between human and robot hopping at the body-to-ground interaction level could be a proof of concept for our proposed mechanical design and motor control model.

### Need for further investigations

 Our robot design and experiments targeted joint mechanics and control and centered on the separation of joint-specific actuation. Adding further inter-joint actuation, e.g., with biarticular GAS PAM, could be one extension that might reveal more details of human hopping. Recently, we examined the effect of VAS and GAS muscles, e.g., in synchronization between ankle and knee joints^62^.

Three locomotor subfunctions can describe locomotion: stance, swing, and balance^34^. This study focused on the *stance* locomotor subfunction, which describes the interaction of the body (CoM) and the ground in the stance phase besides a simple implementation of leg swinging. The proposed model was insufficient to describe the hip function for balancing. One potential extension is employing more detailed neuromuscular models (anchored to templates^28^) to better analyze the role of mechanics and control. Alternatively, we can extend our model to a more realistic morphological design, such as further combinations of mono-and biarticular muscles (e.g., rectus femoris and hamstring muscle) and physiological muscle properties, such as damping effects (in force-velocity relation) to better understand the different locomotor subfunctions and their coordination.

In our robotic setup, the movement of the upper body was constrained to be vertical. As mentioned before, our recently developed EPA-Jumper robot can move freely in the sagittal plane, including nine PAMs representing different muscle groups in the leg. This new setup provides accessibility to investigate the function of mono and biarticular muscles, e.g., thigh biarticular muscles’ role in swing and balance control. These compliant tunable muscles can transfer energy between knee and hip joints. Further, the hip joint actuator function and its control in human hopping and other gaits, e.g., walking and running, can be explored. Finally, the robotic setup needs to be extended to 3D space to investigate other degrees of freedom, especially at the hip joint.

In conclusion, our analysis of human hopping provided new insights into the human joint function and the design and control of the bioinspired EPA-Hopper-II robot. We identified the strategies to generate a specific bouncing movement with the ability to change the gait pace (hopping frequency). This improved understanding was implemented and validated in the robot, giving clear suggestions for improving the design and control. These ideas can be extended to explain other gaits and apply to assistive devices such as prostheses^33^ and exoskeletons^31^.

## Methods

In this section, we first explain the details of human experiments, which follow the mathematical calculation of the passive (spring) and active compliance (FMC) models. Finally, we describe the EPA-Hopper-II setup and the robot experiment protocols.

### Human hopping experiments

The hopping experiments (Fig. 1a) were conducted with six healthy young subjects (6 male, age: $24.1 \pm 3.3$ yrs, mass: $73.5 \pm 6.6$  kg). This study was approved by the Ethical Committee of the Technical University of Darmstadt and was carried out based on the guidelines of the Declaration of Helsinki. All subjects voluntarily provided written informed consent to participate. First, we determined the preferred hopping frequency (PHF) by asking subjects to perform a hopping task at their self-selected hopping frequency and height for 20 s (on both legs) and calculating the PHF for each subject. The mean of PHF for all subjects is $2.5 \pm 0.38$ Hz. After that, each subject performed a series of hopping experiments at different frequencies, 75 %, 100 %, 125 %, and 150 % of PHF. The subjects performed six hopping trials for each hopping frequency, each lasting 20 s. An acoustic metronome guided the frequency in each trial. Between two sequential trials was at least a two-minute resting time. The trials were performed randomly to reduce potential long-term effects (e.g., changes in muscle-tendon unit stiffness, fatigue, etc.).

A total of 20 reflective markers were placed on the following anatomical locations, which were used to determine the kinematics: acromium, anterior and posterior superior iliac spine (ASIS and PSIS), posterior heel, lateral and medial position of the knee, ankle, and toe on both legs. A 10-camera infrared motion capture system (Qualisys, 3+ series, and 6+ series, 500 Hz, Sweden) was used to measure the spatial position of markers. To calculate the GRF and the center of pressure (CoP) of each leg, two force plates (Kistler, Lightweight portable 3D force plate, 1 kHz, Type 9260AA, Switzerland) were used to collect the force data of each leg separately.

OpenSim^63,64^ and its inverse kinematics and inverse dynamics were used to determine the inverse kinematics and dynamics. The kinematic and kinetic data were low-pass filtered at 50 Hz (4^th^ order zero-lag Butterworth).

First, three trials were selected for each subject, in which all kinematic and kinetic data were usable. Then, 20 hops (which is the maximum hops performed by all subjects in all trials) of each of these three selected trials for each subject and for each frequency were chosen. In any case with more hops, 20 hops were selected based on the minimum variation of 7 signals (ground reaction force, joints’ angles, and joints’ torques) from the median of the corresponding signal at that trial. All following movement analyses are then applied to the mean of these 20 hops, which is representative of the trial. In other words, all the calculations in human data are trial-based, and the final result is the mean of all 18 trials (6 subjects $\times$ 3 trials). Finally, to compare the results in different respects, we used one-way ANOVA, and the *p*-values are reported in each section.

### Elasticity coefficient

coefficient $C_{EL}$ introduced in^20^ as an approximation of elastic behavior independent of nonlinearities in spring law is defined as below:

1 $$\begin{aligned} C_{EL}= (1 - A / A_{max})^2 \end{aligned}$$

which is the ratio between the enclosed area by the torque-angle trace of each leg joint (A) and the maximum surrounding squared area of the trace (${A_{max}}$). $A_{max} = \Delta M * \Delta \theta$ where $\Delta M$ and $\Delta \theta$ are the maximum torque and the maximum angular change of the joint, respectively. The coefficient $C_{EL}$ offers a quantification of the degree to which the joint approximates perfectly elastic behavior ($C_{EL} = 1$).

### Determination of passive compliance and motor control

The first step of our analyses is done at the joint level. From the torque-angle patterns (Figure 2) in each joint, we found that a linear spring could be a useful passive elastic element to predict a major contribution of toque generation in different joints.

2 $$\begin{aligned} \tau = k(\theta ^s_0 - \theta ) \end{aligned}$$

Here, $\tau$ and $\theta$ are the joint torque and angle, while *k* and $\theta _0$ are the best approximation of the joint stiffness and rest angle, respectively. We use the least square to find the best-fitted line to the torque-angle behavior (see details of the method in^65^).

The second model for predicting torque generation is the force modulated compliance (FMC)^30^. In this method, we use the following equation:

3 $$\begin{aligned} \tau = CF(\theta ^f_0 - \theta ) \end{aligned}$$

in which *C* and *F* are the stiffness and the normalized ground reaction force (to the subject’s mass), respectively. Here, the *C* and $\theta _0$ will be calculated to find the best approximation of joint torques. The next step is fusing these two approaches of constant (passive) and adjustable (active) compliant elements. For this, we considered a boundary for the spring stiffness and rest angle, which is $\pm 50\%$ of the calculated values in the previous step (using Eq. (2)). Then, we used Eq. (4) to estimate the passive spring and FMC related values.

4 $$\begin{aligned} \tau = k(\theta ^s_0 - \theta )+CF(\theta ^f_0 - \theta ) \end{aligned}$$

Here, the super-indices ^s^ and ^f^ indicate the rest angle of the spring and GRF modulated compliance (FMC), respectively. This hybrid approach needs four parameters to quantify passive mechanical and controlled (force reflex based) terms.

### Robotic experimental setup

EPA-Hopper-II is a one-leg robot designed for studying human-like hopping movements based on human inspired actuation system. This electric-pneumatic actuator (EPA) introduced in Ref.^2^ provides sufficient degrees of freedom to employ electric motors and/or pneumatic artificial muscle (PAM) with a wide range of morphological arrangements. The robot design allows investigation of the role of mechanics and control to generate human-like hopping patterns at different frequencies. This robot, a successor of our previous hopper robots^32,66^, has a 3-segmented leg structure mimicking the human leg morphology in the sagittal plane; Figure 1. Inspired by human hopping experiment analyses, we considered a combination of motor and PAM for knee actuation to replicate FMC+Spring, and only two antagonistic PAMs at the ankle joint. As this robot is constrained to vertical movement at the hip joint, upper body balancing is not an issue; we do not focus on hip actuation in this study. Thus, we only use an electric motor for hip actuation for leg adjustment in the flight phase. Such an actuation allocation is a simple replication of increasing passive to active element roles from distal to proximal joints found in human hopping.

To keep the robot’s weight minimal, the mechanical parts are constructed mainly from 3D printed parts, and the limbs are made of hollow carbon fiber tubes. The whole robot weighs 3.75 kg. The thigh and shank segments of this robot measure 42 and 46 cm, respectively. Its compliant curved foot is designed to mirror the shape of the human foot, contributing to having a natural gait and being more energetically efficient. Additionally, a rubber sheet is affixed beneath the foot to dampen the initial ground contact shock effectively.

The EPA-Hopper-II is redundantly actuated by two brushless direct current electric motors (HYmotor E8318-120KV), one assigned for the direct actuation of the hip and one for the knee actuation using a rope-pulley system with a ratio of 1:5. The motor weighs 0.65kg with a maximum continuous power of 3500W. To circumvent excessive mechanical stiffness and friction within the transmission system, we have opted not to incorporate a gearbox for the motors. Instead, we employ a direct-drive mechanism for the hip and a quasi-direct-drive setup for knee actuation, ensuring transparency between the motors and the external environment. This design choice enables us to attain relatively precise torque control performance through motor current sensing alone, eliminating the need for force/torque sensors. A high-current lithium polymer battery provides the supply source for motors with a capacity of 5000 mAh. Furthermore, the three PAMs, namely TIB (12 cm), SOL (16 cm), and VAS (22 cm), can be used as actuators if needed. The Tibialis and Soleus PAMs are responsible for plantar extension and flexion of the ankle and the Vastus muscle for knee extension. The PAMs are supplied by a JUNAIR piston compressor (Quiet Air 6-15) through continuous valves (PVQ-series solenoid valves). PSE530 pressure sensors are utilized to control the PAM pressures. Although the PAMs control loop (including the sensor and valves) is sufficient to provide sufficient bandwidth for generating hopping (e.g. as used in Ref.^67^,) in this study, we fixed the PAM pressures and used them as passive adjustable compliant elements prescribed by human hopping experiments. The ground reaction force (GRF) is measured by a piezoelectric force plate (Kistler, Lightweight portable 3D force plate, 1 kHz, Type 9260AA, Switzerland). Two encoders (AMT10-series) placed at the hip and knee joint measure the joint angles. Besides, three motion capture cameras (Qualisys, 6+ series, 200 Hz, Sweden) are used to capture the kinematic data of robot hopping.

### Hopping control and implementation

For the control of the robot, the hopping phase is divided into flight and stance phases based on the GRF signal. In the flight phase, the hip and knee joints are position-controlled independently using two PD controllers to prepare the robot for landing in a proper configuration. At the onset of the stance, a 50 ms collision controller is used to transition between the flight and stance controller. This controller serves as a prevention for undesired oscillations at landing and absorption of energy. After this short interval, the control is switched to the force-modulated compliance (FMC) controller while the hip is set free. The FMC uses the vertical GRF measured by the force plate as feedback sensory information to modulate the stiffness of a virtual spring at the knee joint. We use Eq. (3) for the electric motor control. This will be complemented by the parallel PAM to yield Eq. (4) replicating human knee motor control. The ankle joint antagonistic PAMs (SOL and TIB) do not change during hopping.

For finding the control parameters and PAM pressures to generate hopping at 2.5 Hz as the average value for human preferred hopping frequency (PHF), we first conducted a series of optimizations with a simulation model of the robot (See Ref.^62^ for details of the simulation model and optimization approach). Then, we slightly fine-tuned these parameters for the hardware experimental setup to generate hopping at the preferred frequency. To verify the effects of changing the control and mechanical parameters on hopping frequency identified in human experiments, we changed the corresponding values in the robot accordingly. For each hopping frequency, we tuned ankle artificial muscle pressures to specific values to change the stiffness and rest angles as prescribed by human experiments (Fig. 4). We used the patterns found in FMC control values (Fig. 5) to set the knee control parameters for higher and lower frequencies than PHF.

For implementation on the robotic setup, the high-level controllers are implemented in real-time with MATLAB Simulink xPC target. The control command is then sent to the low-level motor drivers. The interface between the xPC target machine and other electronics is through an EtherCAT communication bus at 1 kHz.

The parameters of the robot are not exactly those of humans as they have different body properties e.g., weights. We did optimizations with the simulation model of the robot to generate hopping with a comparable frequency to human PHF (2.5 Hz). Then we fine-tuned By changing the control and PAM parameters inspired by human hopping.

### Supplementary Information

Supplementary Information.

## Data Availability

The processed datasets for all subjects together, along with data on force, inverse dynamics (ID), inverse kinematics (IK), and marker data for each participant used in this study, are available in the TUdatalib^68^. The data from the EPA-Hopper-II hopping experiments used in this study is available in the TUdatalib^68^.
