# Supplementary material for: Role of compliant mechanics and motor control in hopping - from human to robot
Source: Sci Rep. 2024 Mar 21;14:6820. doi: 10.1038/s41598-024-57149-0 (PMC10957903; doi:10.1038/s41598-024-57149-0)
Supplement: Supplementary file 1 — Supplementary Information. [file 41598_2024_57149_MOESM1_ESM.pdf]

Role of compliant mechanics and motor control in hopping - from human to robot
Supplementary Materials

Aida Mohammadi Nejad Rashty[1,*], Maziar A. Sharbafi[1], Omid Mohseni[1], and André Seyfarth[1]

[1]Lauflabor Locomotion Laboratory, Institute of Sport Science and Centre for Cognitive Science, Technical University of Darmstadt, Darmstadt, 64289, Germany
[*]aidamnr@sport.tu-darmstadt.de

ABSTRACT

In this document, we present supplementary results supporting (1) advantages of active control (E.g., using FMC) versus passive springs (including nonlinear springs) in all three joints characterized by residual torque ratio. (2) Work-loop analysis to show the need to have active elements at each joint. (3) Focusing on Torque-angle curves on the knee joint and the ability of the FMC to predict the work-loop, and (4) Demonstrating the importance of ankle stiffness to tune the leg stiffness with a sensitivity analysis and correlation between joint and leg stiffness.

Linear versus nonlinear springs

In the pursuit of exploring various passive compliance models, we evaluated both quadratic and cubic nonlinear springs in addition to the linear spring. When analyzing the second-order nonlinear spring, we observed that the residual torques closely resembled those of a linear spring. However, when we introduced the cubic nonlinear spring, the most significant improvements were observed in the ankle joint, where residual torque ratio ranged from 10-14% down to 4-6%. Additionally, for lower frequencies, the knee joint exhibited a maximum 15% reduction in residuals. Nevertheless, the results were on par with the performance achieved using FMC and FMC combined with a spring.

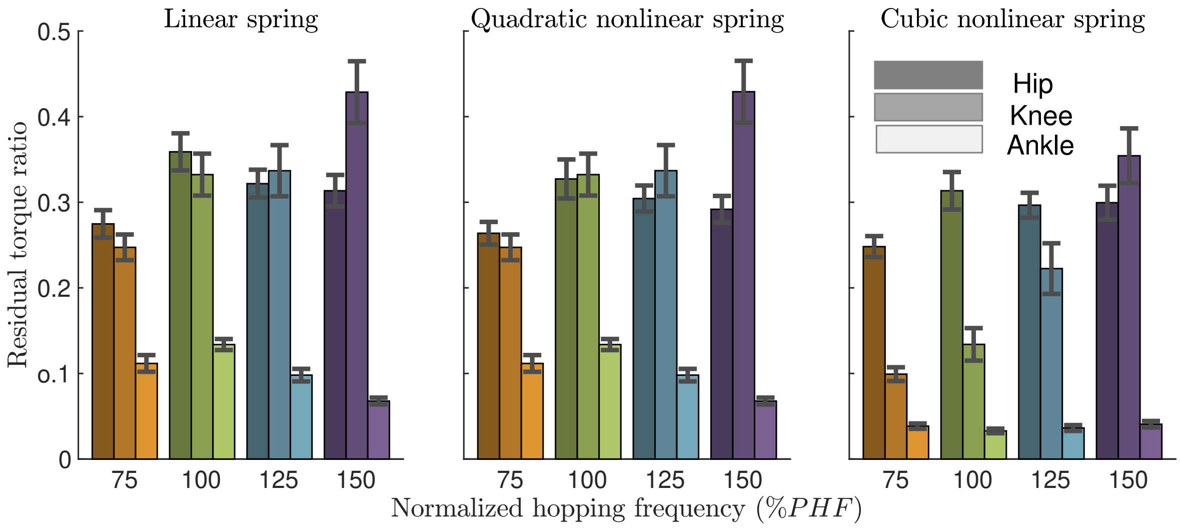

Figure S1. Comparison of residual torque ratio at different joints between linear and nonlinear springs. The ratio of residual torque to the joint torque for different hopping frequencies following the implementation of a linear spring, a quadratic spring, and a cubic spring. The standard error for each value is shown as an error bar.

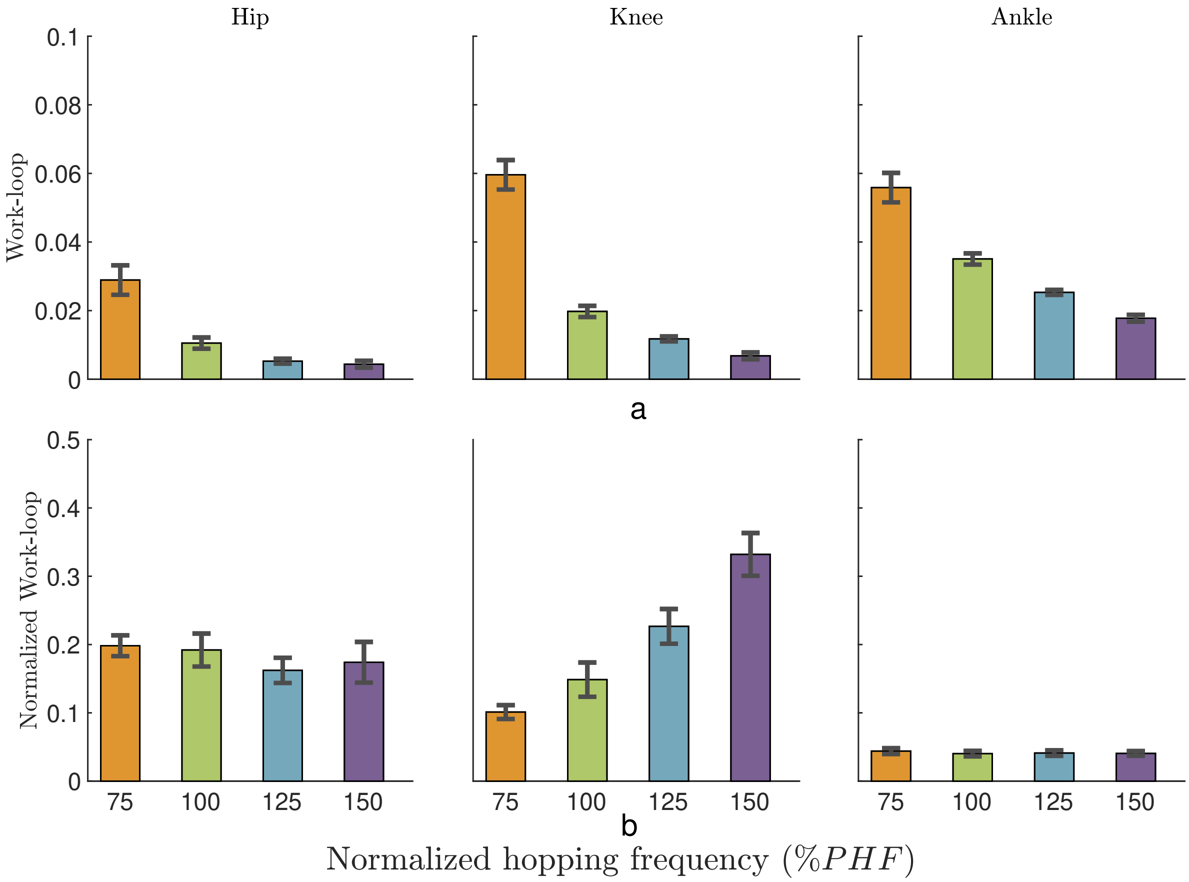

Figure S2. **Work-loop at different joints.** (**a**) The work-loop (WL): the net required (positive and negative) work at the joint, and (**b**) Normalized work-loop is the ratio of WL to the total contribution of the joint in injecting and absorbing energy. The values for four different hopping frequencies and for hip, knee, and ankle are shown. The standard error for each value is shown as an error bar.

Sensitivity Analysis

We developed a simulation model consisting of four segments (foot, shank, thigh, trunk) and three major leg joints to investigate how variations in joint stiffness affect leg stiffness. The segments' lengths were set to the average values found in human experiments. These joints were equipped with constant stiffness springs (ankle, knee, hip). We proceeded by employing the joint stiffness observed in each joint (averaged across all trials) at each frequency and systematically varied the stiffness of one joint while keeping all other parameters constant. We found the resulting leg stiffness at different configurations that the leg accepts in the stance phase of hopping with that specific frequency. We then calculated the average leg stiffness from the model in this movement range for every new combination of joint stiffness. We normalize joint/leg stiffness by dividing their simulation values by the average joint/leg stiffness found in human hopping at corresponding frequencies. Therefore, point (1,1) in each graph represents the human hopping experiment condition in that frequency. Figure S5 illustrates the leg versus joint sensitivity of the leg stiffness to variation of the joint stiffness from 0.5 to 2 times their identified values in human hopping at each frequency. As can be seen, leg stiffness exhibits a high sensitivity (p-value < 0.05) to alterations in ankle joint stiffness while remaining relatively insensitive (p-value > 0.05) to variations in knee or hip stiffness. This suggests that the observed changes in ankle stiffness among the subjects were the most significant mechanism for adapting leg stiffness, which can be seen in correlation coefficients (Table S1).

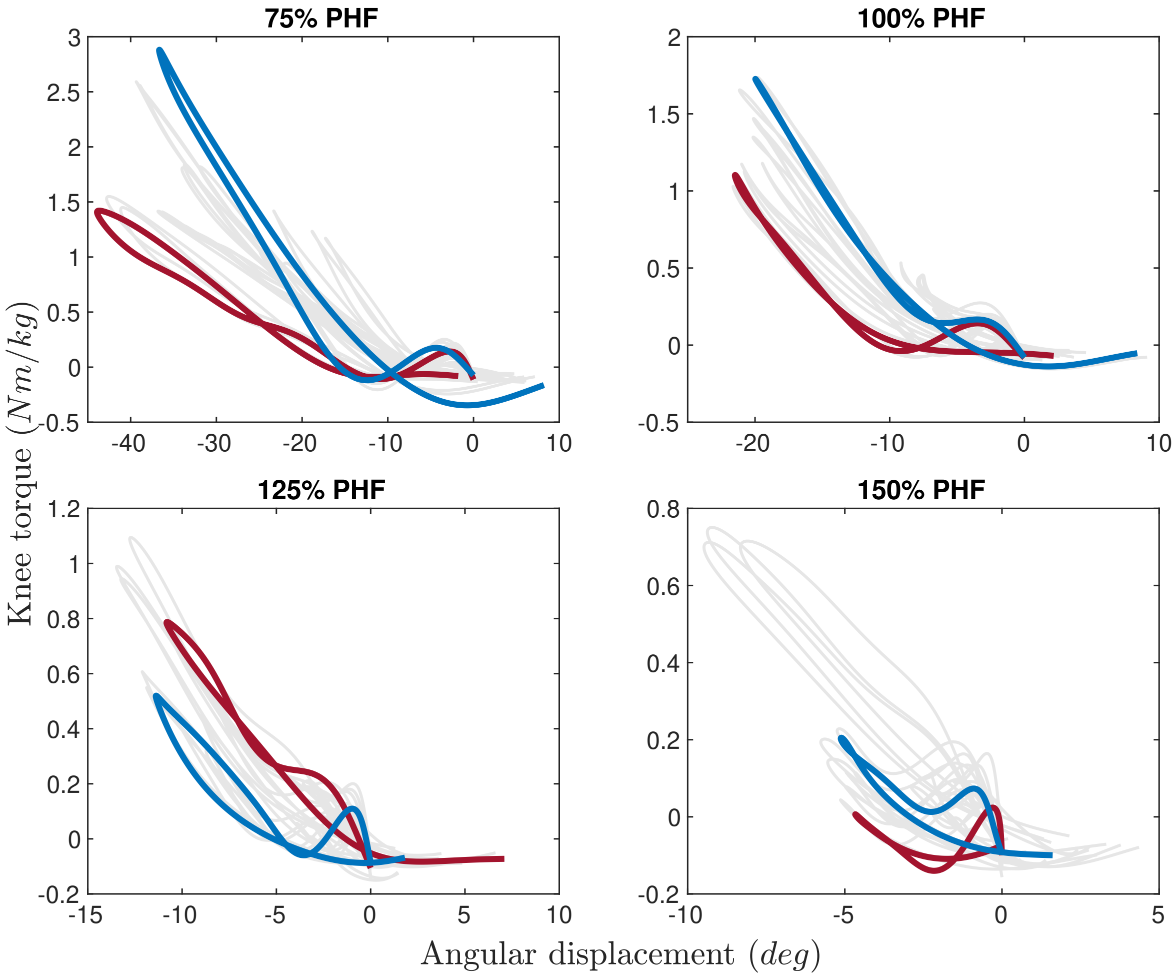

Figure S3. **Knee work-loop diagrams.** The torque-angle relation for the knee joint in different frequencies for all 18 trials is shown in light grey, while two typical behaviors at each frequency are depicted in blue and red. Negative and positive work-loops are observed, showing the insufficiency of the passive elements to replicate the knee behavior and the necessity of active energy management at the knee joint.

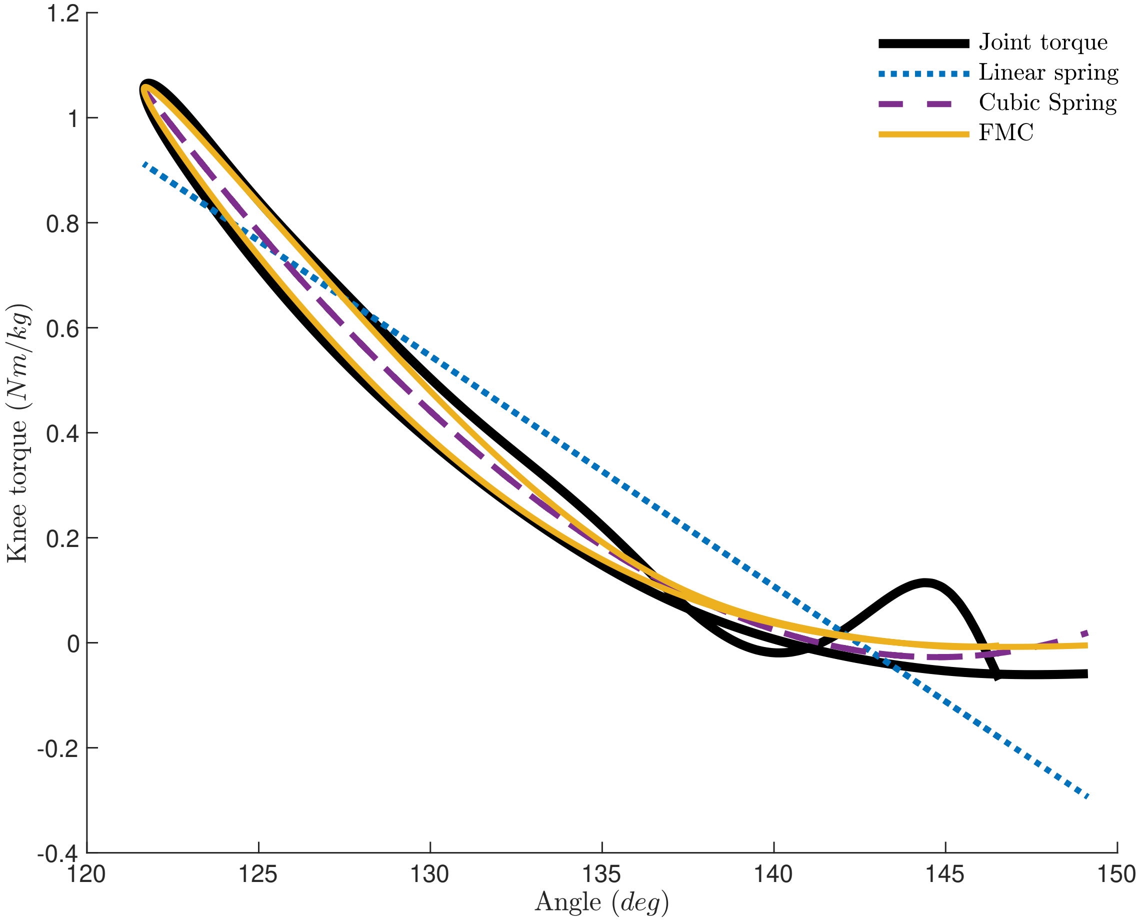

Figure S4. Estimation of the knee torque-angle relation with different methods. The results of three models are shown: linear spring (dotted line), cubic spring (dashed line), and FMC model. It shows that the FMC model could nicely predict the work-loop, which is not possible with passive springs. The knee joint torque for one trial of 75 %PHF is shown as an example.

Joint	Frequency (% of PHF)	correlation	p-value
Hip	75	0.19	0.45
	100	-0.02	0.93
	125	-0.1	0.69
	150	0.2	0.48
Knee	75	0.49	0.04
	100	-0.65	0.0032
	125	-0.72	0.0007
	150	-0.59	0.01
Ankle	75	0.75	≈ 0
	100	0.86	≈ 0
	125	0.84	≈ 0
	150	0.54	0.02

Table S1. Correlation coefficient between leg stiffness and each joint stiffness. The calculated correlation coefficients between hip, knee, and ankle joints with leg stiffness for different frequencies and the corresponding p-values are reported.

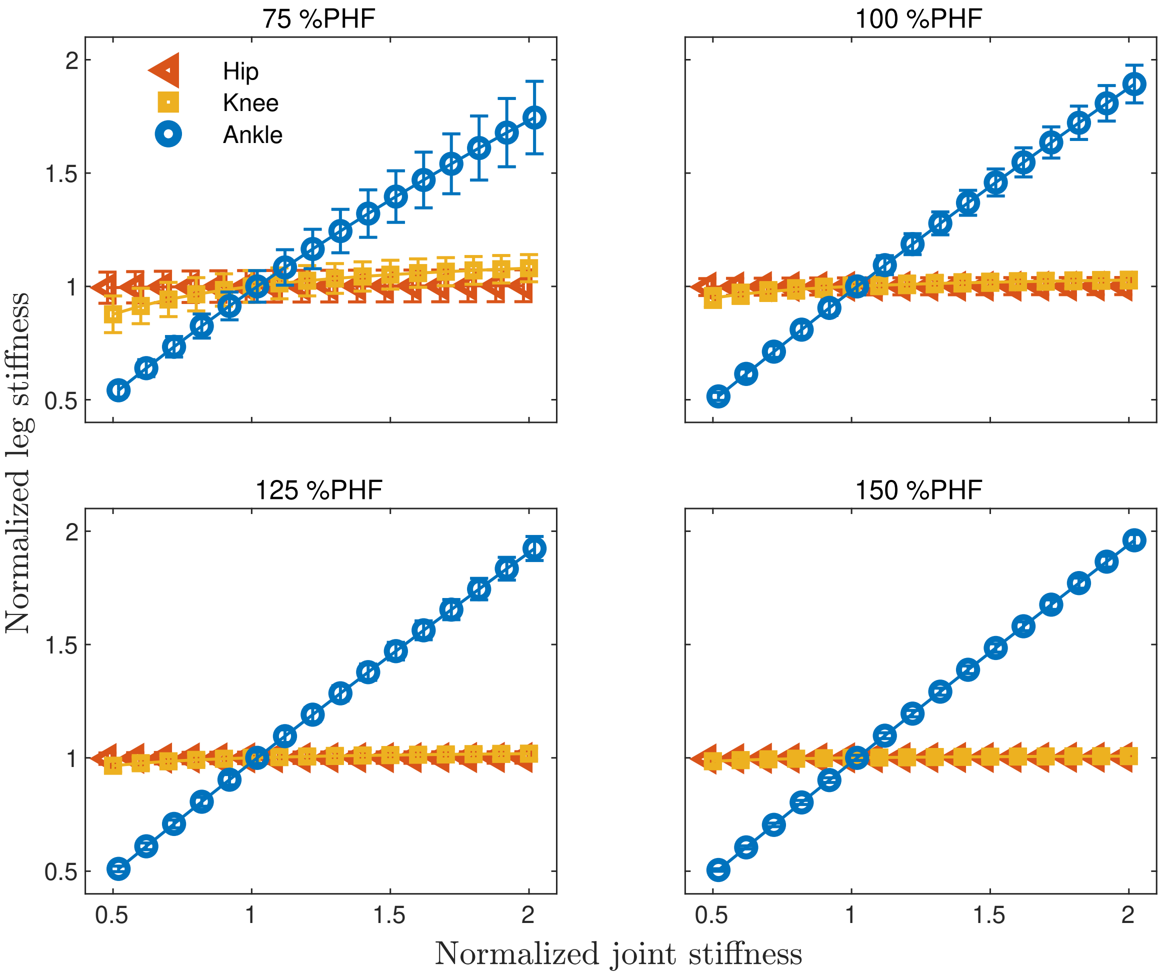

Figure S5. Sensitivity of the model's leg stiffness to changes in joint stiffness. A 4-segmented simulation model with human hopping experimental data at different frequencies is used.
